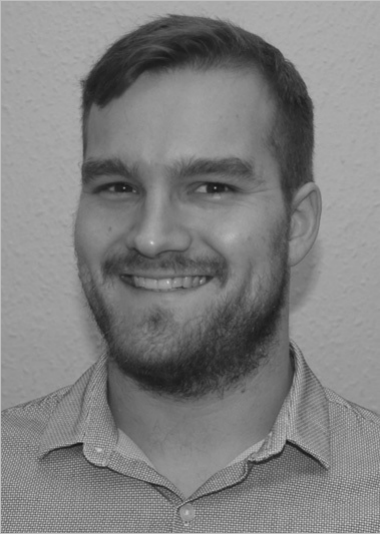# Molecular Knots

**DOI:** 10.1002/anie.201702531

**Published:** 2017-08-16

**Authors:** Stephen D. P. Fielden, David A. Leigh, Steffen L. Woltering

**Affiliations:** ^1^ School of Chemistry University of Manchester Oxford Road Manchester M13 9PL UK

**Keywords:** interlocked molecules, molecular knots, supramolecular chemistry, template synthesis, topology

## Abstract

The first synthetic molecular trefoil knot was prepared in the late 1980s. However, it is only in the last few years that more complex small‐molecule knot topologies have been realized through chemical synthesis. The steric restrictions imposed on molecular strands by knotting can impart significant physical and chemical properties, including chirality, strong and selective ion binding, and catalytic activity. As the number and complexity of accessible molecular knot topologies increases, it will become increasingly useful for chemists to adopt the knot terminology employed by other disciplines. Here we give an overview of synthetic strategies towards molecular knots and outline the principles of knot, braid, and tangle theory appropriate to chemistry and molecular structure.

## 1 Introduction

**Figure 1:**
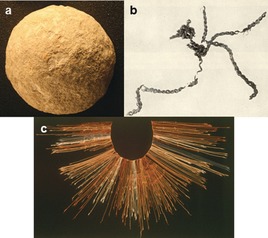
The impact of knotting on technology: a) Spherical stones thought to be bola weights, which would need to be tied together for hunting, date back 500 000 years.^1^ b) The oldest surviving man‐made knots are those of the Antrea net, a fishing net made of willow with a 6 cm mesh dating to 8300 BC.^2^ c) Knots, in the form of quipu, have been used to record and communicate information, with the earliest examples possibly predating the invention of the written word.^3^ Image (a) “Stone ball from a set of Paleolithic bolas” reproduced from https://goo.gl/vyAh85 (downloaded 5 May 2017) under a wikimedia creative commons license. Image (b) “Pieces of the Antrea net” reproduced from https://goo.gl/y0026E (downloaded 5 May 2017) under a wikimedia creative commons license. Image (c) “Quipu from the Inca Empire” reproduced from https://goo.gl/tqZyPW (downloaded 5 May 2017) under a wikimedia creative commons license.

Knots are basic elements of structure that are exploited in tools, materials, architecture, and construction.^1^ In prehistory, the ability to tie knots had a major impact on human development, enabling early man to make useful implements such as bolas, axes with blades tied to handles, and fishing nets,^2^ and eventually to weave fabrics. Knots have even been used by some civilizations to store and pass on information^3^ (Figure 1).

**Figure 2:**
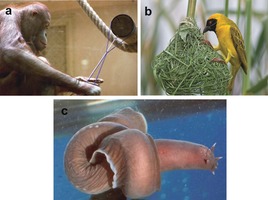
Knotting exploited by animals: a) Wattana the orangutan tying a knot.^4^ b) The African weaver bird uses knots to tie its nest securely.^5^, ^6^ c) Hagfish knot their bodies to generate force when pulling flesh off a carcass.^7^ Image (a) reproduced from Ref, 4 with permission from the University of Chicago Press. Image (b) “southern masked weaver by wim de groot” reproduced from https://goo.gl/ZpD09h (downloaded 5 May 2017) under a wikimedia creative commons license. Image (c) reproduced from Ref. 7 with permission from Springer Nature.

Humans are not the only species to use knots. Other primates have been observed to tie knots to make tools (Figure 2 a).^4^ Some birds, a spectacular example being the weaver bird, incorporate knots into their nests^5^, ^6^ (Figure 2 b), and hagfish and some eels tie themselves into knots as a mechanism to generate leverage when tugging at flesh^7^ (Figure 2 c).

The physical significance of knotting is increasingly becoming apparent in fields as varied as colloids,^8^ liquid crystals,^9^ optical beams,^10^ soap films,^11^ superfluids,^12^ and the origins of the early universe.^13^ In molecular terms, knots are found in circular DNA^14^ and approximately 1 % of proteins,^15^ and they form spontaneously in polymer chains of sufficient length and flexibility.^16^ As every sailor, mountaineer, and scout knows, different types of knots have different characteristics that make them more or less suited for a particular task: “bend knots” give the strongest binding between two lengths of rope; “hitches” are best for tying rope around an object; and “loop knots”, or “nooses”, allow degrees of movement between the components they connect.^17^ There is no reason to suppose that different types of knots will not be just as important, versatile, and useful in the molecular world. However, scientists will not be able to investigate that hypothesis until they are able to access a significant range of different molecular knot topologies.

The rigorous mathematical study of knots began in the 19th century as an attempt to explain atomic theory. Peter Guthrie Tait's initial forays into knot theory were carried out at Lord Kelvin's suggestion that in doing so he might find evidence to support the theory that atoms were knotted vortices in the “lumniferous aether”, with each element corresponding to a different knot.^18^, ^19^ The “knotted aether” theory was short‐lived,^20^ but interest in the classification and mathematical properties of knots continued. With the discovery, and ultimately the synthesis, of molecular knots in the latter part of the 20th century, knot theory and chemistry share a close relationship once again. Here we give an overview of the current state‐of‐the‐art in the synthesis and properties of molecular knots and how their structures relate to broader knot theory.

## 2 Knot Theory

A knot is mathematically defined as a circle embedded in 3D space. Different knots are, therefore, different entanglements in a closed loop, rather than in the open strings in which we find knots in our everyday world. The “closed‐loop” definition is necessary from a topological standpoint as any entanglement in a linear strand with two ends can be untied by deformation (for example, the untying of shoelaces). The following section gives an overview of knot theory and terminology relevant to chemistry and molecular structure.

### 2.1 Representations of Knots

**Figure 3:**
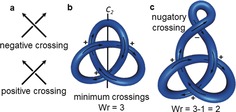
Reduced diagrams and writhe: a) Definition of a negative and a positive crossing. b) A knot can be oriented by following its loop in an arbitrary direction. In the depicted orientation, the shown trefoil knot has a writhe (Wr) of 3, as all three crossings are positive. The orientation can be reversed by rotation along the indicated *C_2_*‐axis. c) In a trefoil knot with nugatory crossings, the writhe can take any value. Knot diagrams without nugatory crossings are referred to as “reduced”.

The minimum number of crossings, where one string passes over or under another, is one of a knot's “invariants” (that is, an intrinsic property of a particular knot). The simplest representation of a knot, often referred to as the “reduced representation”, is one depicting the fewest number of crossings. Further crossings can be introduced by twisting the knot, a conformational change in molecular structural terms. Such twists are called “nugatory crossings”.^21^ Each knot can be represented in an infinite number of different representations by adding nugatory crossings to the reduced representation. For example in Figure 3 c, a nugatory crossing has been added to the reduced form of the trefoil knot (3_1_) that has three crossings in Figure 3 b.

A knot can be oriented, which means that an arbitrary point of the knot is chosen and the entire loop of the knot is traversed in a given direction. In an oriented knot, positive and negative crossings can be distinguished (Figure 3 a). Positive crossings describe a right‐handed helix, negative crossings a left‐handed helix. This allows another property to be quantified, the writhe (Wr) of a knot diagram. Writhe is the sum of positive and negative crossings, where a positive crossing has a value of +1 and a negative crossing has a value of −1. Writhe is not a property of the knot, but of the knot diagram (in other words, a particular conformation of a molecular knot). From a topological standpoint, writhe can take any value, as any number of positive or negative nugatory crossings can be added; in practice the restrictions on conformations that can be adopted for a small‐molecule knot will limit writhe (nugatory crossings will generally add conformational strain and be entropically unfavorable unless stabilized in some way). For most knots the writhe of the reduced representation is independent of the reduced representation chosen (an exception being the so‐called “Perko pair”, see Section 2.9).

**Figure 4:**
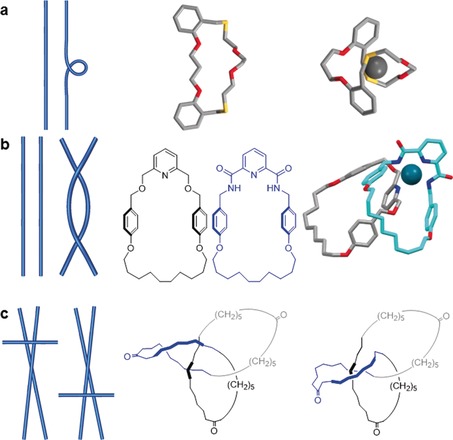
Reidemeister moves and examples of their (supra)molecular equivalents. Reidemeister moves allow transitions between any two diagrams (i.e. conformations) of the same knot or link. They are named after the number of components involved in the movement. a) Reidemeister I refers to the creation or removal of a nugatory crossing, the number of crossings/writhe changes by ±1. It is equivalent to twisting a macrocycle to form an additional loop.^24^ b) Reidemeister II passes one string over another, the number of crossings changes by ±2 but the writhe remains the same. It is equivalent to moving one molecular strand over another.^25^ c) Reidemeister III refers to passing a string over a crossing. The number of crossings and writhe are unchanged. Note the nugatory crossing in the left‐hand triketone trefoil knot structure, which is necessary for a Reidemeister III move for any alternating knot.^26^

To move between any different diagrams of a knot, a combination of just three different types of manipulations are necessary, known as the Reidemeister moves (independently discovered by Reidemeister^22^ as well as Alexander and Briggs^23^ in the 1920s). These moves are only applied to a small section of the knot and are characterized by the number of strands involved (Figure 4). The Reidemeister I move describes the addition of a nugatory crossing to a single strand, and is the only Reidemeister move which changes the overall writhe of the knot (Figure 4 a). A chemical example would be introducing a twist in a macrocycle. The Reidemeister II move moves one loop entirely over another, thereby creating two crossing points (Figure 4 b) and corresponding in chemical terms to moving one macrocycle over another, for example. The Reidemeister III move involves three strands: one is moved over the crossing of two others, a transformation represented by conformational changes within a molecular trefoil knot (Figure 4 c).

**Figure 5:**
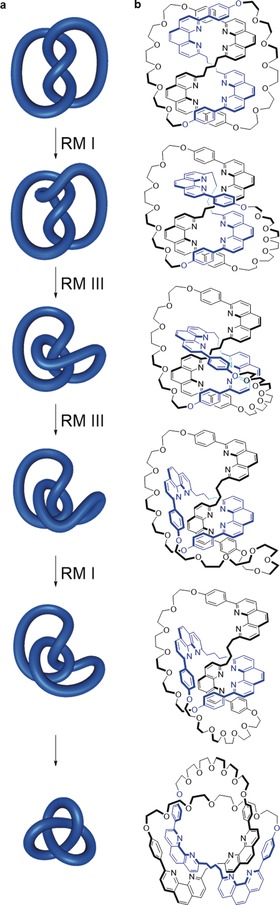
Reidemeister moves converting different representations of a trefoil knot into each other: a) A trefoil knot is converted from a *D*_2_‐symmetric double helix to a *D*_3_‐symmetric form. b) The same process (corresponding to conformational changes in a molecular structure) applied to Sauvage's trefoil knot^27^ (in this case molecular *D*_3_‐symmetry cannot be achieved as one loop is chemically different to the other two).

In Figure 5 a Reidemeister moves are used to transition between the *D_2_*‐symmetric and *D_3_*‐symmetric representations of a trefoil knot; Figure 5 b shows the same process for a molecular knot, whereby the conformation in which Sauvage's original molecular trefoil knot^27^ is synthesized is transformed through the linear helicate approach (Section 3.1.1) to the conformation in which molecular trefoil knots are synthesized using single metal‐ion templates (Section 3.1.2).

### 2.2 Classification of Knots

A convenient way to classify knots is using the Alexander–Briggs notation,^23^ commonly used for knots with up to 10 crossings and used throughout this Review. In this notation, every knot is denoted in the form *X_Y_*, where *X* corresponds to the number of crossings and *Y* is an index that distinguishes the knot from others with the same number of crossings. *Y* was originally determined by sorting the knots for a given number of crossings by increasing torsion number. The torsion number is a mathematical property of a knot that was popular in the first half of the 20th century.^28^

The most basic characteristic of a knot is whether or not it has crossings, that is, whether a knot is trivial or nontrivial. A trivial knot has no crossings and can be deformed to a torus. A trivial knot is sometimes referred to as the “unknot”, denoted 0_1_, and in molecular terms corresponds to a macrocycle.

### 2.3 Prime and Composite Knots

**Figure 6:**
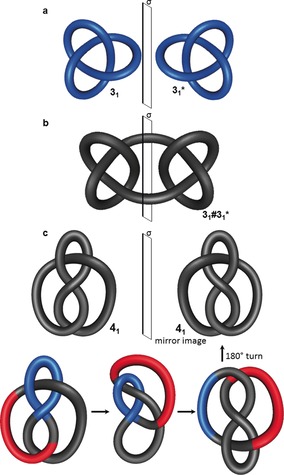
Chirality in knots. a) The trefoil knot 3_1_ is topologically chiral, as it cannot be deformed to its mirror image form 3_1_* without the strand passing through itself. b) The square knot 3_1_#3_1_*, a composite knot obtained by connecting two trefoil knots of opposite handedness, is achiral, as the mirror plane σ projects it onto itself. c) The figure‐eight knot 4_1_ can be transformed into its mirror image. By flipping the part shown in red over the part shown in blue, an upside‐down version of the mirror image is obtained after deformation, thereby making it topologically achiral. For the sake of brevity, not every Reidemeister move is shown here for this transformation.

Nontrivial knots have at least three crossings and are either prime or composite. Prime knots cannot be constructed by combining simpler knots, whilst composite knots can, by performing the so‐called “knot sum”.^29^ This is analogous to numbers: prime numbers can only be divided by themselves and one, composite numbers are the product of smaller prime factors. Composite knots are written using the hash symbol (#) to connect the Alexander–Briggs notation of the constituent prime knots (using +/− to indicate the handedness of each prime knot if the handedness is defined, or * to indicate the opposite handedness of a prime knot compared to the others if only relative handedness is relevant, as in Figure 6).

The number of knots with the same number of crossings increases dramatically with an increasing number of crossings: there are 2 prime knots with 5 crossings, 165 with 10 crossings, and more than 250 000 with 15 crossings. There is no known formula to calculate the number of possible prime knots for a given number of crossings.^21^

### 2.4 Chirality in Knot Theory

A chemical compound is chiral if its structure cannot be projected on to itself by a rotary reflection and cannot be deformed to its enantiomer due to a sufficiently high inversion barrier. This is sometimes referred to as Euclidean chirality. Mathematically, however, knot projections can be deformed as much as desired (without the strand passing through itself) and knots are only topologically chiral if they cannot be deformed continuously to superimpose with their mirror image. Thus, objects with Euclidean chirality are not necessarily topologically chiral (e.g. molecules with asymmetric carbon centers or the mirror images of the reduced representation of the figure‐eight knot 4_1_ shown in Figure 6 c). The trefoil knot 3_1_ is topologically chiral, as one mirror‐image form cannot be continuously deformed into the other (Figure 6 a). Although this is clearly true from simple observation, the topological chirality of a trefoil knot was only proven mathematically in the early 20th century.^30^

Most knots are topologically chiral; of the more than 1.7 million prime knots with up to 16 crossings, fewer than 2000 are achiral. Achiral knots were initially referred to as “amphichiral” or “amphicheiral” (a term introduced by Tait) by mathematicians, but over the time the term “achiral”, so familiar to chemists, has also become common in topology.^31^

An example of a topologically achiral knot is the composite square knot, which is formed from the knot sum of two trefoil knots with opposing handedness, and has a plane of symmetry where the knots are joined (Figure 6 b). The representation of the topologically achiral 4_1_ knot in Figure 6 c appears to be chiral at first sight, as it is not immediately apparent how the knot can be deformed to its mirror image. However, through a series of Reidemeister moves, one mirror‐image form can be converted into the other, thereby demonstrating that the 4_1_ knot is topologically achiral.

**Figure 7:**
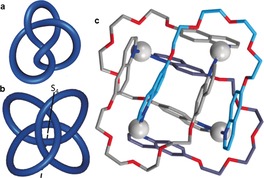
Geometric representations of a topologically achiral knot. a) At first sight the reduced representation of the 4_1_ knot does not look achiral. b) However, the introduction of nugatory crossings enables the 4_1_ knot to adopt an achiral conformation possessing an *S*_4_ axis. c) A coordination complex with the 4_1_ knot topology (see Scheme 15).^33^

The absence of chirality in the 4_1_ knot is easier to see if a more symmetrical representation^32^ is used. Figure 7 a shows the 4_1_ knot in its reduced form with four crossings, while a more spherical form with eight crossings is shown in Figure 7 b. The spherical representation is formed by adding four nugatory crossings to the reduced representation and has a rotary inversion axis (*S*_4_), which means that a rotation of 90° converts this representation into its mirror image, thereby making it achiral. Figure 7 c shows a coordination complex^33^ with the same spatial arrangement of ligand strands (see Scheme 15 for its synthesis and chemical structure).

### 2.5 Invertible Knots

Some knots are invertible, which means that they can be continuously deformed to give a reversed orientation of the closed loop. If the trefoil knot is oriented as in Figure 3 b, the orientation changes when the knot is rotated along one of its *C*_2_ axes. The existence of non‐invertible knots was only discovered in 1963, as most knots with low crossing numbers are invertible (the simplest non‐invertible knot is 8_17_).^21^ For higher numbers of crossings, the fraction of non‐invertible knots rises dramatically. In topology, chiral invertible knots are termed “reversible”, chiral non‐invertible knots are termed “fully chiral”, and achiral invertible knots are referred to as “fully achiral”.^21^

### 2.6 Alternating and Non‐Alternating Knots

**Figure 8:**
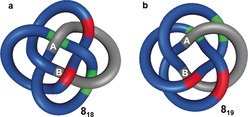
Alternating and non‐alternating knots. a) 8_18_ is an alternating knot, as overpasses (red) and underpasses (green) alternate (over‐under‐over‐under etc) around the entire length of the strand, as exemplified along the gray path from A to B. b) 8_19_ is a non‐alternating knot (over‐over‐under‐under etc), shown on the gray path from A to B. The 8_19_ knot cannot be represented by a solely alternating crossing pattern.

A striking feature of knots with fewer than 8 crossings is that they can all be represented in forms in which overpasses and underpasses alternate when the strand is traversed (Figure 8 a). Knots that can be represented this way are said to be alternating. It was originally thought that all knots can be drawn in an alternating pattern, but the existence of non‐alternating knots was demonstrated by Little^34^ and proven during the 20th century.^35^, ^36^, ^37^ Figure 8 b shows one of the three simplest non‐alternating knots, 8_19_. All alternating achiral knots have an even number of crossings.^21^ In addition, all reduced representations of an alternating knot have a constant writhe. This is not necessarily the case for non‐alternating knots, which historically led to some duplications in knot tables.^38^, ^39^ If an alternating pattern is achieved for a knot, it can generally be easily determined whether the knot is prime and distinguished from others, this is harder for non‐alternating knots. Although alternating knots are dominant for knots with low crossing numbers, the fraction of alternating knots appears to tend exponentially towards zero when the crossing number is increased.^21^

### 2.7 Torus Knots

**Figure 9:**
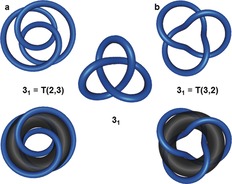
Torus knots. A torus knot T(*p*,*q*) runs *p* times in the poloidal direction (i.e. through the cavity) and *q* times in the toroidal direction (i.e. around the cavity) around the surface of a torus without the strand intersecting. Swapping *p* and *q* results in the same knot. a) T(2,3) is the trefoil knot 3_1_. b) T(3,2) is also the trefoil knot.

Torus knots are a family of knots that can be drawn on the surface of a torus (donut shape) without the closed loop intersecting itself. They can be abbreviated by the symbol T(*p*,*q*), where *p* and *q* are integers that describe how many times the torus is passed in the poloidal and toroidal^40^ directions, respectively, before the two ends are joined. A torus knot is obtained if *p* and *q* are co‐prime.^41^ Switching *p* and *q* gives the same torus knot with a different geometry, as shown for the trefoil knot in Figure 9. All torus knots are topologically chiral (except if *p* or *q*=1, which yields the unknot). For knots with an odd number of crossings, the knot of lowest order in the Alexander–Briggs notation X_1_ is always a torus knot. Torus knots are amenable to chemical synthesis by linear (Section 3.1.1) and circular (Section 3.3.2) double helicate approaches.

### 2.8 Twist Knots

**Figure 10:**
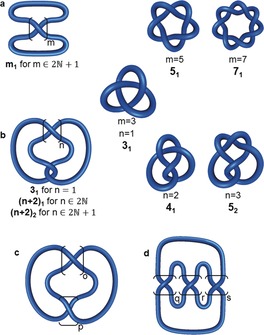
Generation patterns for torus, twist, clasp, and pretzel knots. a) For odd values of *m*, a torus knot *X*_1_ is obtained (*X* is the number of crossings). These knots are generated by twisting two strands around each other. For even values of *m*, two component links are created. b) This construct gives a twist knot for any positive *n*. Twist knots with an even number of crossings are denoted as *X*_1_, those with an odd number as *X*_2_ (in this case X_1_ is a torus knot). c) Twist knots are a type of clasp knot C(*p*,*o*). Twist knots are C(2,*o*) clasp knots. d) The generation pattern for (*q*,*r*,*s*) pretzel knots. Pretzel knots consist of left or right‐handed helices connected together (see Section 2.11).

Twist knots are another family of knots that are generated by a defined pattern: two strands are twisted *n* times and the open ends linked before closure. This process is illustrated in Figure 10 b. For an even number of crossings, X, the twist knot is X_1_ in Alexander–Briggs notation. For odd numbers of crossings, the twist knot is X_2_ (X_1_ is in this case a torus knot). Especially for low numbers of crossings, twist knots are very abundant, three of the four simplest knots are twist knots (the trefoil knot 3_1_, the figure‐eight knot 4_1_, and the three‐twist knot 5_2_). Topoisomerases tend to form predominantly twist knots in DNA, as these topologies result when the topoisomerase breaks a DNA duplex at a node and allows the crossing duplex to pass through the gap before resealing the broken DNA.^42^

Twist knots are a subset of a larger group of “clasp knots” (Figure 10). The general structure of a clasp knot C(*p*,*o*) is shown in Figure 10 c. For twist knots *p*=2.^43^

### 2.9 Knot Tables

**Figure 11:**
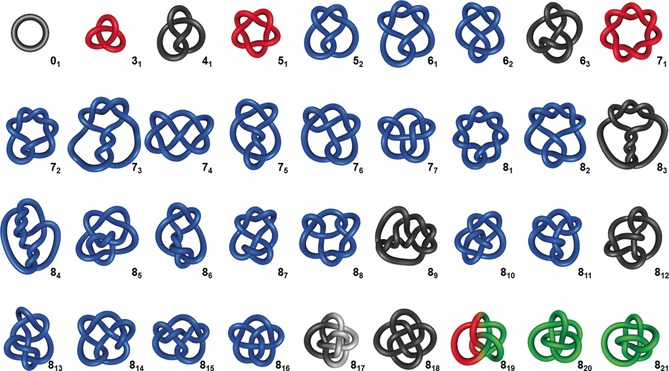
Knot table of all prime knots having up to eight crossings including the unknot 0_1_. Torus knots are depicted in red, achiral knots in black, non‐invertible knots in white, and non‐alternating knots in green.

The systematic tabulation of knots started in the 19th century,^18^, ^19^ one of the most commonly used versions today is the Rolfsen knot table.^44^ All knots (alternating and non‐alternating) with up to 16 crossings have likely been found,^21^ with the number standing at slightly more than 1.7 million prime knots. The last pair of duplicates (two structures in knot tables that are actually the same knot) to be discovered, was two representations of a 10‐crossing knot called the “Perko pair” in the 1970s.^39^ The tabulation of alternating knots has been extended up to 22 crossings, with more than 6 billion found to date.^29^ Figure 11 shows a knot table with all prime knots having up to eight crossings.

### 2.10 Braid Representations

**Figure 12:**
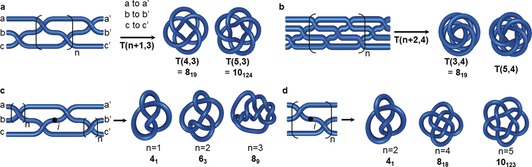
Braid representations of knots. a) A braid for the generation of three‐strand torus knots. A knot is generated from the corresponding braid by connecting opposite ends without generating additional crossings. b) This braid generates torus knots with one additional toroidal revolution for each extra value of *n*. Following this pattern with additional strands in the braid, any torus knot can be obtained. c) A braid for the generation of a family of achiral knots. For any number of *n*, a reverse rotated palindrome (RRP) is obtained, indicated by the inversion center *i*. d) This braid also generates achiral knots for any number *n* not divisible by 3.

A braid is a set of discrete strands that cross each other in a defined pattern. To create a corresponding closed‐loop knot, the ends of the strands are connected so that no additional crossings are generated, as illustrated in Figure 12. Every knot can be represented as a braid and, therefore, for chemists, a braid indicates a potential synthetic pathway to any given molecular knot topology. The pattern for the simplest torus knots in Figure 10 a consists of two strands twisted about each other with the ends connected. Braid representations of higher order torus knots are shown in Figure 12 a,b.

Figure 12 c,d show braid representations for several achiral knots. Note that the braids have an inversion center (indicated with a dot, *i*, in the figure). Such braids are called reverse rotated palindromes (RRP),^45^ and if a knot can be represented by an RRP then it must be achiral. The braid in Figure 12 c forms the achiral 4_1_ knot (*n*=1), repeating the recurring unit (*n*=2) gives the achiral 6_3_ knot and repeating it once more (*n*=3) leads to the achiral 8_9_ knot. The braid shown in Figure 12 d is an RRP for any value of *n* and is also a “Brunnian braid”, as removal of any one strand leaves the other two unconnected.^46^, ^47^

Just as a knot has an infinite number of diagrams, a knot can also be represented by an infinite number of different braids. However, every knot has a minimum braid representative, analogous to the reduced representation of a knot. The minimum braid is the one with the fewest number of crossings and strands. An advantage of braid representations over traditional knot diagrams is that they can be conveniently stored in computer‐readable form.^48^

### 2.11 Tangle Representations^49^

Knots can be broken down into smaller key fragments, so‐called “tangles”, which were introduced by Conway^50^ and have proved useful in describing the behavior, properties, and transformations of local entanglements.

**Figure 13:**
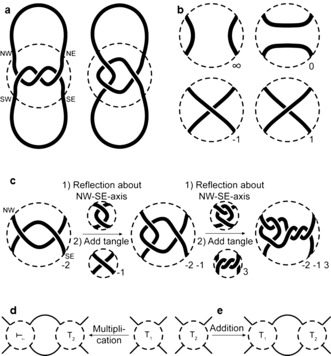
Tangle representations. a) A section of a knot (indicated by a dashed circle) can be split into tangles. The fixed entry points of the string are named after the cardinal directions. b) Some basic tangles for the construction of more complex tangles. c) For the construction of a rational tangle, the starting tangle is reflected along the NW‐SE axis and the new tangle added to the NE and SE crossing points. The resulting tangle can be further extended by the same procedure. d) Generalization of tangle multiplication. T_2_ is not restricted to being an integer tangle such as in (c). e) A different way to connect tangles is by addition. The sum is formed by connecting the NE and SE crossing point of the first tangle T_1_ to the NW and SW crossing point of the second tangle T_2_, respectively.

A tangle is a region of a knot that can be surrounded by a circle so that the knot crosses the circle exactly four times. The crossings over the circle are fixed and named after the points of a compass: NW, NE, SW, SE (Figure 13 a). The Reidemeister moves described in Section 2.1 can be performed on tangles. Although the two knots shown in Figure 13 a are equivalent (see Figure 5) the two tangles are not (the strands run either from NW to SE or from NW to SW).

Tangles can be constructed from basic building blocks, some of which are shown in Figure 13 b. A tangle with two parallel strands running from NW to SW and NE to SE is called the ∞ tangle. Two parallel strands running from NW to NE and SW to SE, gives the 0 tangle. A tangle containing a positive crossing is a 1 tangle, and one with a negative crossing is a −1 tangle. These building blocks can be combined (multiplied) to give rational tangles, as illustrated in Figure 13 c. Starting with a −2 tangle (two negative crossings), the tangle is first reflected along the NW‐SE axis. This is then joined to a second tangle, which in this case is a −1 tangle, thereby resulting in a −2 −1 tangle. To add a third tangle, the original tangle is reflected along the NW‐SE axis and then the additional tangle added (in this case a 3 tangle) to obtain a −2 −1 3 tangle. If the ends of a rational tangle are connected, a rational knot (or link) is obtained, such as for Figure 13 a, where a trefoil knot is formed.

This process can be further generalized with tangle multiplication: not only integer tangles (such as the −1 tangle, 2 tangle, etc.) can be connected in this way, so can more complicated ones. This is illustrated in Figure 13 d. Furthermore, there is the operation of tangle addition, as shown in Figure 13 d. These operations are not necessarily commutative or associative. The resulting tangles are called algebraic tangles and can be closed to form algebraic knots.

Tangle addition leads to a diagram that is sometimes referred to as a “pretzel knot” (*q*,*r*,*s*), as shown in Figure 10 d. The integers *q*, *r*, and *s* define either right‐handed (positive values) or left‐handed helices. For example, the trefoil knot can be described as the (1,1,1) pretzel knot. Changing the sign of all descriptors yields the other enantiomer of the same knot.^51^ The first non‐invertible knots that were discovered belonged to the class of pretzel knots.^51^

Tangles are a convenient way to describe and classify even complex knots in terms of how the knot is structured locally. The knotted regions in the knots shown in Figure 10 can be described as tangles, and every torus knot is obtained by closing an *m* tangle (Figure 10 a), while every twist knot is obtained by closing an *n* 2 tangle (Figure 10 b). Just as braids can be seen as a strategic blueprint for synthetic chemists for constructing different knots, tangles provide a way of thinking about synthons for crossings that need to be assembled in a particular way.

### 2.12 Interconversions of Knots

**Figure 14:**
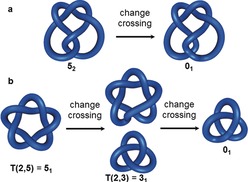
Unknotting numbers. a) Any twist knot has an unknotting number of 1, as inverting one crossing is sufficient to give the unknot 0_1_. b) The unknotting number of a torus knot T(*p*,*q*) is 1/2 (*p*−1)(*q*−1), In this example, the pentafoil knot 5_1_ is converted into a trefoil knot 3_1_ by inverting one of its crossings. Changing a second crossing gives the unknot 0_1_. So the unknotting number is 2 (=1/2 (2−1)(5−1)).

Knots can be transformed into other knots by inverting (i.e. removing or adding) crossings. One characteristic of knots is their “unknotting number”, which refers to the minimum number of crossings that have to be inverted to give the unknot from a given knot. The unknotting number for some knots can be easily determined: the unknotting number of a twist knot is always 1 (Figure 14 a) and for a torus knot T(*p*,*q*) is 1/2 (*p*−1)(*q*−1) (Figure 14 b).^52^ The unknotting number is often more difficult to determine for other knots,^49^ but it is always less than half of the total number of crossings for a given knot.^53^

**Figure 15:**
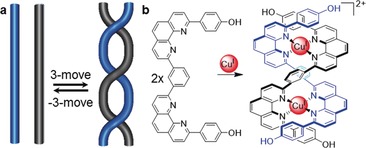
Changing entanglement using *k*‐moves. a) A *k*‐move introduces *k*‐positive crossings in a set of two strings, a −*k*‐move introduces *k*‐negative crossings. b) A supramolecular 3‐move induced by Cu^I^ ions forms the scaffold for the synthesis of a molecular trefoil knot.^27^

One way of transforming a knot is to change its tangles (Section 2.11). Another possibility is to use so‐called *k*‐moves.^49^ A *k*‐move describes the introduction of *k* positive (or −*k* negative) crossings into a set of two strands (Figure 15 a). This is related to the way chemists introduce crossing points into molecules by, for example, using metal‐ion coordination to twist, orientate, or entwine ligand strands upon binding (Figure 15 b).

How closely two knots are related can be expressed by the “Gordian distance”, that is, the number of crossing changes needed to interconvert the knots.^54^ It can be seen in Figure 14 b that inverting one crossing of the pentafoil (5_1_) knot yields the trefoil (3_1_) knot. The Gordian distance between the two knots is thus 1.

## 3 Synthesis of Molecular Knots

The synthesis of molecular knots requires mechanical restriction of the relative positions of molecular components in a similar manner to that needed to construct other mechanically bonded molecular architectures, namely links (catenanes) and threaded molecular rings (rotaxanes).^55^, ^56^ It is, therefore, unsurprising that many of the advances in the synthesis of small‐molecule knots have come from groups also active in catenane and rotaxane synthesis. Successful syntheses of small‐molecule knots have been reported since the late‐1980s.^56^, ^57^, ^58^, ^59^, ^60^ In the following section the most significant methods and strategies for the synthesis of small‐molecule knots developed to date are discussed.

### 3.1 Molecular Trefoil Knots (3_1_)

**Scheme 1:**
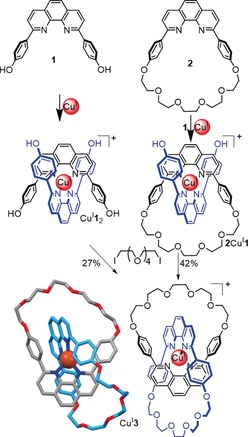
Sauvage's synthesis of a [2]catenane (Cu^I^ **3**) by passive^62^ metal‐template synthesis. The phenanthroline‐Cu^I^ system formed the basis for the synthesis of several other mechanically interlocked molecular types (rotaxanes, trefoil knot, Solomon link).^61^ All of the cap‐and‐stick structures shown in this Review are X‐ray crystal structures produced from coordinates taken from the Cambridge Structural Database (CSD).

The trefoil knot (3_1_) is the simplest nontrivial knot and the most amenable to chemical synthesis. Many different methods to synthesize trefoil knots have been reported. The first of these was Jean‐Pierre Sauvage's linear metal helicate strategy, an extension of the method his group employed in the metal‐template synthesis of [2]catenane (Hopf link)^61^ Cu^I^ **3** (Scheme 1). The tetrahedral Cu^I^ ion holds the two bidentate ligands in a mutually orthogonal arrangement such that the curvature of the ligands creates two crossing points. The metal ion can subsequently be removed after macrocyclization to yield metal‐free [2]catenane **3**. The synthesis can be carried out using one preformed macrocycle (**2**) or having both ligands (**1**) macrocyclize around the template (Scheme 1).

#### 3.1.1 Linear Double Helicates

**Figure 16:**
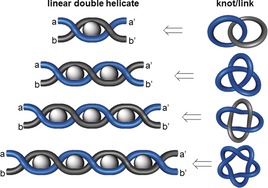
The linear helicate approach to simple knots and links. Metal ions induce the twisting of the ligand strands to form a double helix (*k*‐moves, Section 2.12). If the number of crossings is odd, a molecular knot is created upon connecting a/a’ and b/b’. For an even number of crossings, links ([2]catenanes) are produced. The linear helicate approach was successfully demonstrated by Sauvage for the first three in this series (Hopf link, trefoil knot, and Solomon link), but fails for higher order topologies such as the pentafoil knot 5_1_ (Section 3.3.1).

**Scheme 2:**
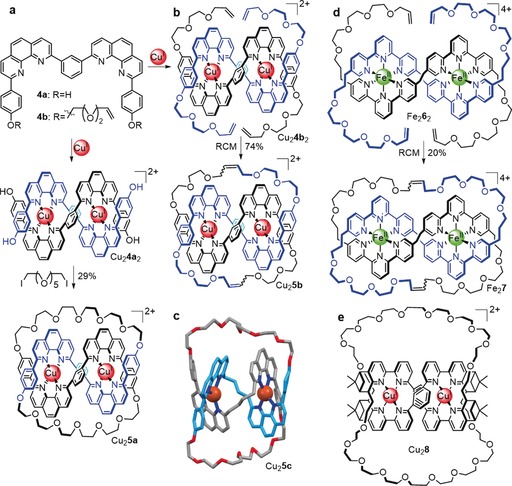
Molecular trefoil knots prepared from a linear helicate strategy. a) Synthesis of trefoil knot Cu_2_ **5 a** after covalent capture of linear double helicate Cu_2_ **4 a**_2_ by Williamson ether synthesis.^64^ b) The yield of the trefoil knot was significantly increased by using RCM for the macrocyclization reactions.^65^ c) X‐ray structure of the related trefoil knot Cu_2_ **5 c**. This early design was obtained in lower yield, as the alkyl chain connecting the phenanthroline units is less preorganized than the *m*‐phenylene unit used in later designs.^63^ d) A related approach using Fe^II^ and terpyridine derivatives instead of Cu^I^ and phenanthroline ligands.^66^ e) Enantioselective synthesis of a trefoil knot by the linear helicate approach.^68^

Sauvage realized that this metal‐template approach could be extended to form linear helicates that produce more complicated interlocked structures on closure (Figure 16). Such linear double helicates can be considered a chemical system based on the braid for the synthesis of T(*x*,2) torus knots shown in Figure 10 a. The next topology to be synthesized after the [2]catenane was the trefoil knot 3_1_, obtained from a dinuclear Cu^I^ complex.^27^ The molecular topology was determined unambiguously by X‐ray crystallography^63^ (Scheme 2 c). In initial designs, the phenanthroline units were connected by short alkyl chains and the resulting yields of the trefoil knot were low (<10 %). Introduction of a *m*‐phenylene unit increased the preorganization of the helicate, and designs based on ligand **4 a** gave trefoil knot **5 a** in almost 30 % yield^64^ (Scheme 2 a). Ring closing metathesis (RCM) as a method of covalent capture increased the yield of the knot to 74 % (**5 b**; Scheme 2 b).^65^ The template system could also be varied: the use of octahedral Fe^II^ and two tridentate ligands **6**, instead of Cu^I^ complexes of bidentate ligands, yielded molecular trefoil knot **7** 66 after RCM (20 % yield).

As noted in Section 2.4, trefoil knots are chiral. The enantiomers of **5 a** could be separated by cocrystallization with a chiral phosphate anion.^67^ The use of enantiopure ligands allowed the synthesis of a single enantiomer of trefoil knot **8**, thereby demonstrating the influence of geometric chirality on topological chirality for the first time in a molecular knot.^68^

#### 3.1.2 Single Metal Ion Templates

**Figure 17:**
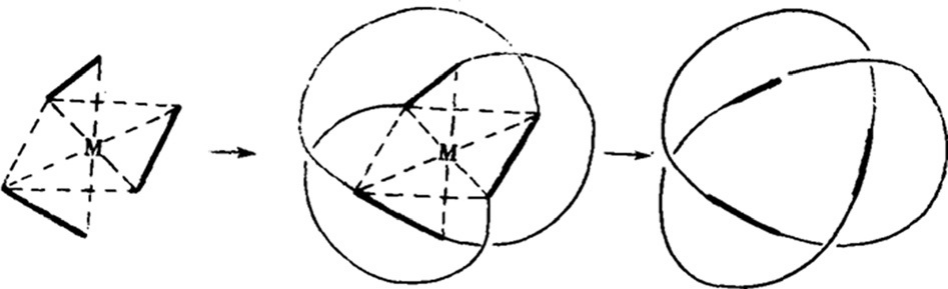
Sokolov's proposed route for the synthesis of a molecular trefoil knot templated by the octahedral coordination sphere of a transition metal. Modified from Ref. 69 with permission from the Royal Society of Chemistry.

**Scheme 3:**
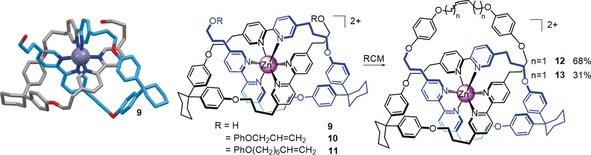
Hunter's synthesis of a trefoil knot using a single metal ion template, via open knot Zn**9**. Functionalizing the ends of the open knot with alkene units enabled closure to the trefoil knot by RCM. The structure of the open knot was determined by X‐ray crystallography.^70^, ^71^

The idea of using transition metal ion templates to assemble catenanes and knots actually predates the first Sauvage catenane by a decade. In 1973 Sokolov proposed^69^ that octahedral metal ions might be used to position three ligands in mutually orthogonal orientations suitable for the template synthesis of a trefoil knot (Figure 17). Nearly 30 years later Hunter and co‐workers prepared an “open knot” by wrapping a single ligand strand containing three bipyridine units around an octahedral Zn^II^ ion with bisphenol Z derivatives as a bend‐inducing linker (Scheme 3).^70^ The “overhand” knot Zn**9** could then be closed by RCM to give trefoil knot **12** or **13**, depending on the length of the chain employed.^71^

**Scheme 4:**
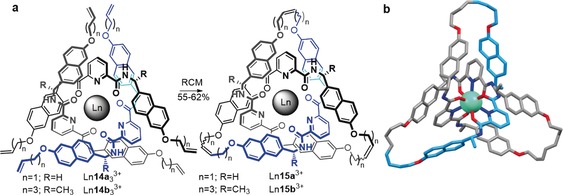
Synthesis of a trefoil knot by the circular helicate approach. a) A single lanthanide ion entwines three 2,6‐diamidopyridyl ligand strands **14** in its coordination sphere, thereby forming a trefoil knot upon connection of the ligand end groups. The achiral precursor **14 a** yields a racemic mixture of the two enantiomers of trefoil knot Ln**15 a**. The use of *C*_2_‐symmetric ligand **14 b** gives enantiopure trefoil knot Ln**15 b**. b) X‐ray crystal structure of enantiopure trefoil knot Ln**15 b**.^72^, ^73^

**Scheme 5:**
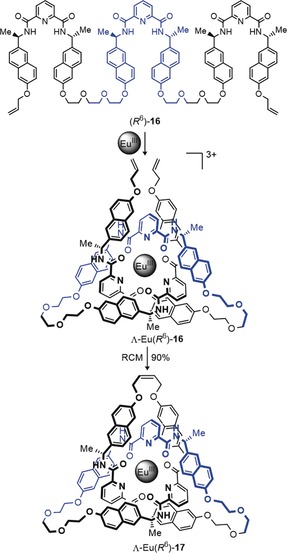
Synthesis of an enantiopure knot from single ligand strand **16**. The addition of Ln salts generates an open knot complex of single handedness which can be closed to the trefoil knot **17**.^74^

A particularly short and efficient synthesis of trefoil knots can be achieved using lanthanide ions as the template (Scheme 4). A europium or lutetium trication was used to assemble a circular trimeric helicate from three 2,6‐diamidopyridyl ligands (Scheme 4 a). Joining the ligand ends by RCM afforded trefoil knot **15 a** in 55–62 % yield.^72^ Introducing chiral centers into the ligand strands gave a trefoil knot (**15 b**) of single handedness^73^ (Scheme 4 a). The X‐ray crystal structure of the enantiopure knot is shown in Scheme 4 b. The use of a single ligand strand **16** incorporating three 2,6‐diamidopyridyl units reduces the number of closures required to from the knot from three to one, thereby enabling knot **17** to be obtained in up to 90 % yield (Scheme 5).^74^

#### 3.1.3 Active Metal Template Synthesis

**Scheme 6:**
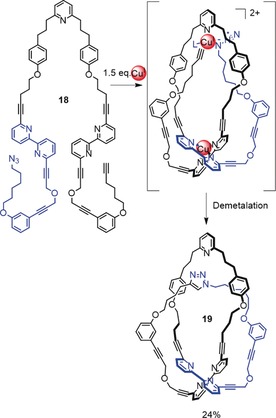
Active template synthesis of trefoil knot **19**. One crossing is generated by Cu^I^ coordination to the bipyridine groups, which forms a loop. A CuAAC reaction of the azide and alkyne termini is directed through the loop by the second coordinated Cu^I^ ion, thereby forming the trefoil knot.^78^

Active template synthesis^55^ is a strategy for forming mechanically interlocked molecules, whereby metal ions play an active role in catalyzing the bond‐forming reactions that covalently capture the final product as well as organizing the building blocks in the manner of a conventional “passive” template. The approach was originally introduced to facilitate the synthesis of rotaxanes^75^, ^76^ and catenanes.^77^ However, the concept has been successfully extended to the synthesis of a trefoil knot (Scheme 6).^78^ Ligand **18**, which possesses one pyridyl and two bipyridyl units, binds a Cu^I^ ion between the two bipyridyl units to create a crossing point. A copper‐catalyzed alkyne–azide cycloaddition (CuAAC) reaction of the azide and alkyne termini through the resulting loop by a second Cu^I^ ion coordinated to the pyridine group generates the other two crossings required for the trefoil knot. ^1^H NMR and drift tube ion mobility mass spectrometry (DT IM‐MS) studies demonstrated that the reaction product **19** had the topology of a trefoil knot.

#### 3.1.4 Directing Trefoil Knot Formation through π‐Interactions and/or Hydrogen Bonding

**Scheme 7:**
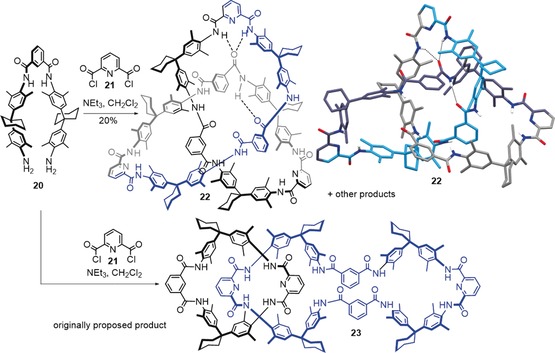
The condensation product of **20** and **21** was originally proposed by Hunter to be [2]catenane **23**.^81^ Several years later, X‐ray crystallography by the Vögtle group showed that the product was actually trefoil knot **22**.^82^ A network of hydrogen bonds responsible for directing the assembly of the knot is shown by dashed lines.

The Stoddart group utilized π–π interactions to try to direct the assembly of a trefoil knot through the macrocyclization of a single strand, although the putative knot was isolated in <1 % yield and proved difficult to fully characterize.^79^ Hydrogen bonding is the directing influence for the formation of several trefoil knots formed as unexpected reaction products. The first of these was a compound first isolated by Hunter, who isolated a compound expected from previous work^80^ to be an amide [2]catenane **23** with two rings of different size^81^ (Scheme 7, bottom). The Vögtle group repeated the synthesis several years later and obtained an X‐ray crystal structure that showed that this compound was actually a trefoil knot (**22**; Scheme 7, top).^82^ It later proved possible to separate the two knot enantiomers.^83^ This episode illustrates the important role that X‐ray crystallography can play in unambiguously identifying a particular molecular topology; it is all too easy to misinterpret ^1^H NMR spectroscopy and mass spectrometry data with complex, often highly symmetrical, knot and link architectures.

**Scheme 8:**
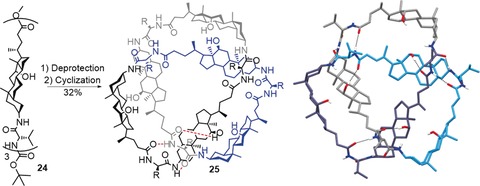
A trefoil knot **25** obtained by ring closure of steroid trimer **24**. An extended network of hydrogen bonds is visible in the solid‐state structure, which has almost perfect *C*_3_‐symmetry (the hydrogen bonds are indicated by dashed black bonds in the crystal structure and as red dashed lines in one of the subunits in the diagram).^84^

Hydrogen bonding was the driving force behind the assembly of another organic trefoil knot based on steroid‐derived building blocks, serendipitously discovered by Feigel^84^ (Scheme 8). In contrast to **22**, which is rather unsymmetrical in its X‐ray crystal structure because of the hydrogen‐bonding network (Scheme 7), trefoil knot **25** shows almost perfect *C_3_* symmetry in the solid state. As a consequence of the chirality of the building blocks, the synthesis of **25** is enantioselective and yields only a trefoil knot of Δ‐handedness.

#### 3.1.5 Dynamic Combinatorial Libraries

**Scheme 9:**
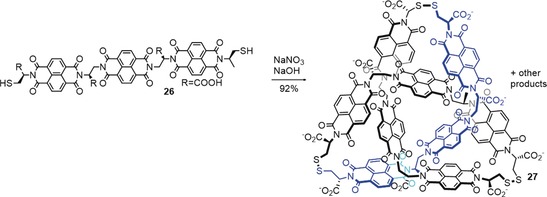
A molecular trefoil knot **27** discovered in a dynamic covalent library. The interlocked structure minimizes the exposure of the hydrophobic surface area to the solvent by burying part of the molecule in the central cavity.^87^

Dynamic combinatorial chemistry (DCC) is a powerful tool for generating interchanging mixtures of compounds, so‐called dynamic combinatorial libraries (DCLs).^85^, ^86^ In recent years, knots have been found in DCLs that generate cyclic oligomers (sometimes also including catenanes) of various sizes. Sanders and co‐workers discovered that trefoil knot **27** was formed in a DCL built from building blocks of the trimeric electron‐poor π‐system **26** (Scheme 9).^87^ By using water as the solvent and dynamic disulfide exchange to establish the DCL, trefoil knot **27** could be formed in high yield. The driving force for knot formation is likely the minimization of the hydrophobic surface area in the knotted structure.

**Scheme 10:**
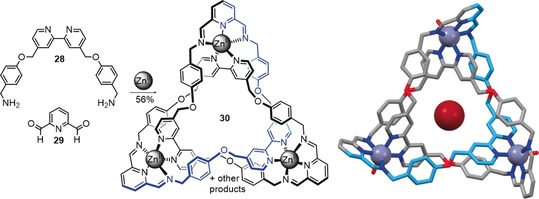
Synthesis of trefoil knot **28** based on imine exchange.^88^ During the crystallization process, two bromide anions are incorporated in the central cavity, one above the other.^90^ Attempts at crystallization in the absence of Br^−^ were unsuccessful.

Trabolsi and co‐workers also discovered a trefoil knot unexpectedly formed in a dynamic mixture of building blocks,^88^ similar to a system used by the Stoddart group to assemble Borromean rings.^89^ Dynamic imine bond formation between diformylpyridine **29** and bipyridine **28** in the presence of Zn^II^ to template the assembly process produced trefoil knot **30** along with a Hopf link catenane and Solomon link (Scheme 10).^88^ The solid‐state structure of **30** features two Br^−^ ions, one above the other, in the trefoil knot cavity (Scheme 10).^90^ The imine groups of the knot could be subsequently reduced with NaBH_4_ if Cd^II^ ions were used as the template in the knot‐forming reaction.^91^

#### 3.1.6 Covalent Scaffolds and Statistical Approaches

**Scheme 11:**
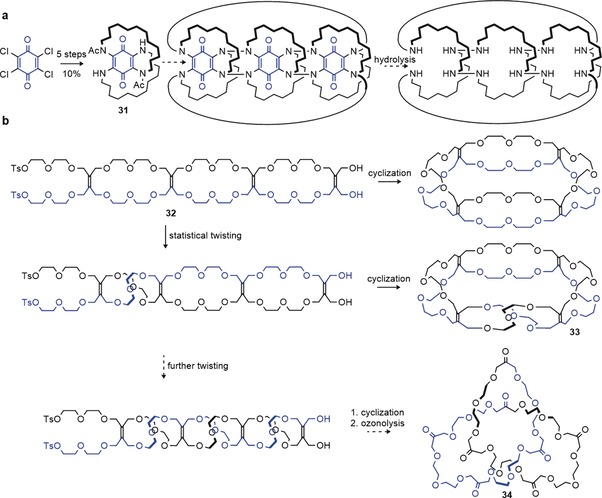
a) Schill's approach towards a molecular trefoil knot using a covalent scaffold. Trimerization of a crowded quinone **31** was projected to give a molecular trefoil knot after cyclization and hydrolysis.^92^ b) Walba's Möbius strip approach towards molecular knots. Introducing a half twist in compound **32** before connecting the ends yields molecular Möbius strip **33**. Statistical twisting was too disfavored to yield trefoil knot **34** after ozonolysis.^93^

One of the first proposed synthetic approaches towards molecular trefoil knots was suggested by Schill and Tafelmair through the use of a crowded quinone **31** (Scheme 11). Trimerization of such a quinone and subsequent cyclization could yield a trefoil knot upon hydrolysis (Scheme 11 a). In practice, the synthetic route was too long to be realized.^92^ It should be noted that it is crucial to connect quinones of the same handedness, otherwise the crossings can be removed through a Reidemeister II move.

An alternative approach by Walba et al. used ethylene bridges between two glycol chains (Scheme 11 b).^93^ It was hoped that such a glycol chain of sufficient length **32** would statistically twist around its own axis, thereby giving a molecular knot **34** after cyclization and ozonolysis of the alkene rungs. Although it was possible to induce one half‐twist by this approach, thus producing a molecular Möbius band^94^ **33**, multiple twists were too disfavored to yield knotted products.

**Scheme 12:**
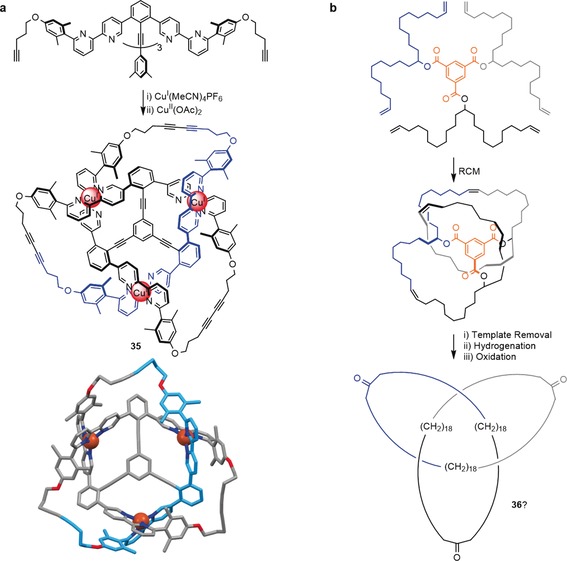
Towards the synthesis of molecular knots by using covalent scaffolds. a) A hybrid approach towards a molecular trefoil knot by using Cu^I^ and a 1,3,5‐substituted benzene as the template. The interlocked structure of **35** after cyclization was confirmed by X‐ray data, but it was not possible to remove the central benzene template to yield a knot.^96^ b) A trefoil knot assembled around a benzene‐1,3,5‐tricarboxylic acid template. The template could be removed after cyclization, but the formation of **36** could not be confirmed experimentally.^26^

Other covalent scaffold approaches toward trefoil knots include Siegel's hybrid approach in which a 1,3,5‐trisubstituted benzene together with three Cu^I^ ions acts as a template for the synthesis of an interlocked species **35** (Scheme 12 a). Removal of the central benzene unit from the structure would generate a trefoil knot.^95^, ^96^

Fenlon assembled a polyethylene trefoil knot around a benzene‐1,3,5‐tricarboxylic acid template and used RCM to close the knot. Although the template could be removed, insufficient characterization data was obtained to confirm the formation of trefoil knot **36** (Scheme 12 b).^26^

#### 3.1.7 Metallaknots

**Scheme 13:**
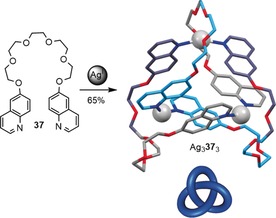
Synthesis of a metalla‐trefoil knot by trimerization of ethylene glycol bridged quinolines **37** with Ag^I^. Coordination bonds from oxygen to silver are omitted for clarity.^33^

Hosseini and co‐workers have described the synthesis of several knotted molecular structures with metal centers as integral parts of the topology, termed metallaknots.^33^ Strictly speaking, these structures are not true knots, as the metal center is coordinated to other parts of the molecule and such branching is not within the definition of a knot as a closed loop. Other examples of branched knotted molecular systems include ravels^97^ and knotted cages.^98^ Scheme 13 shows the synthesis of a trefoil metallaknot based on Ag^I^ coordination to a ligand consisting of two quinoline units bridged by an ethylene glycol oligomer.

### 3.2 Molecular Figure‐Eight Knots 4_1_

**Scheme 14:**
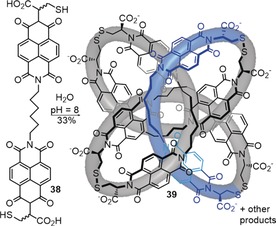
Synthesis of molecular figure‐eight knot **39** by disulfide exchange in a dynamic covalent library. Knotting likely results from the hydrophobic effect.^99^

Although a significant number of synthetic routes, both accidental and designed, have now been established for the simplest knot (trefoil 3_1_), examples of the neighboring entry in knot tables (Figure 11), the figure‐eight knot (4_1_), are scarce. So far, the reduced representation with four crossings has not been realized as a small molecule. However, the eight‐crossing representation of the 4_1_ knot with an *S*_4_‐axis (Figure 7) has likely been discovered in a DCL (its structure determination based largely on symmetry and NMR data).^99^ Similar to the related trefoil knot (Scheme 9), the driving force for the formation of the 4_1_ knot (**39**) is minimization of the hydrophobic surface area, as the synthesis was carried out in an aqueous buffer (Scheme 14). Although a figure‐eight knot is topologically achiral (see Section 2.4), knot **39** is chiral due to the cysteine moieties present in the chain. A *meso* form of compound **39** was also prepared.

**Scheme 15:**
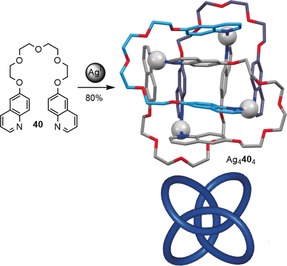
Synthesis of a figure‐eight metalla‐knot by tetramerization of ethylene glycol bridged quinolines **40** with Ag^I^. Coordination bonds from oxygen to silver are omitted for clarity.^33^

Currently, the only other example of a molecular figure‐eight knot is a metallaknot described by Hosseini and co‐workers (Scheme 15).^33^ The crystal structure features an intricate array of π‐interactions and the coordination geometry of the Ag ions with the glycol units and quinoline moieties is responsible for the formation of the metallaknot. The 4_1_ metallaknot also adopts the eight‐crossing *S*_4_‐symmetrical knot representation.

### 3.3 Molecular Pentafoil Knots 5_1_

#### 3.3.1 Linear Helicate Approach

**Scheme 16:**
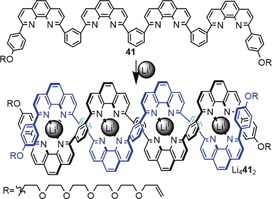
Synthesis of a linear double helicate with five crossings. Attempts to ring‐close Li_4_ **41**_2_ to the corresponding pentafoil knot (5_1_) were unsuccessful.^97^

The figure‐eight knot (4_1_) is followed by the pentafoil knot (5_1_) in knot tables (Figure 11). Similar to the trefoil knot, the pentafoil knot is a torus knot (Section 2.8). This suggests that the linear double helicate strategy (Figure 16) might be suitable to form such a knot. The Sauvage group was able to form a trinuclear linear helicate and close it to the corresponding Solomon link.^100^ However, all attempts to synthesize a pentafoil knot from a tetranuclear linear helicate Li_4_ **41**_2_ failed (Scheme 16).^101^

**Figure 18:**
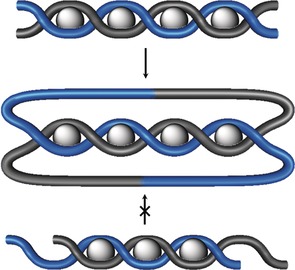
Incorrect registry of ligands disfavors formation of a desired topology. In addition to an increased probability of connecting the wrong strand ends of a linear helicate, partly interwoven helices can become kinetically trapped with longer ligands.

There are likely several reasons for the failure of this strategy. Firstly, as the helicate becomes longer, the distance increases between the strands of the braid that need to be closed to give the desired product, so incorrect closures become more likely. In addition, the center of longer helicates can be significantly strained, so it is likely that some mismatched helices also form (Figure 18).

#### 3.3.2 Circular Helicate Approach

**Figure 19:**
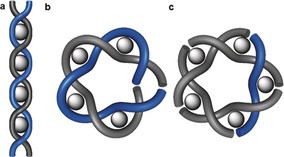
Transition from a linear double helicate to a circular double helicate. a) One of the limiting factors of the linear helicate approach is the increasing distance between the ends. b) Bending the helix brings the ends closer together, but does not reduce the length (and complexity) of the ligand strands. c) In a circular helicate, an additional metal ion brings additional organization to the ends of the helix. The symmetry enables shorter (simpler) ligands to be used at the cost of requiring more reactions (five as opposed to two for the linear helicate in the case of a 5_1_ knot) to achieve closure of the loop.

The inherent problems of linear helicates for the synthesis of knots (and links) can be overcome by bringing the ends of the helicate closer to each other through a bent or fully circular design (Figure 19). The high symmetry of a circular helicate also means that simpler ligands can be used, thus making the chemical synthesis easier, as fewer recognition motifs per ligand are required. However, the number of new bonds that need to be generated to form the closed‐loop knot increases, which suggests that reversible bond‐forming reactions that can “error check” the assembly process could be advantageous.

**Scheme 17:**
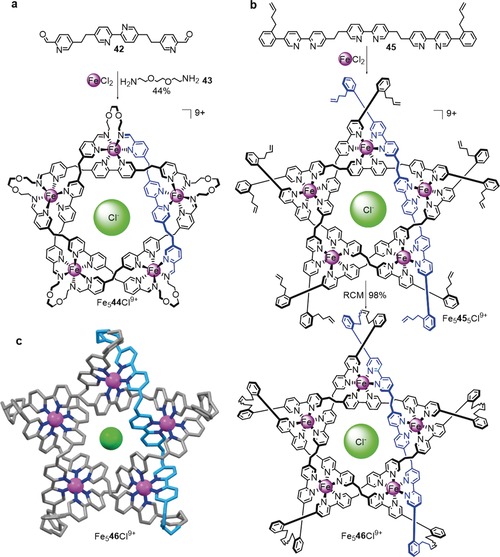
Synthesis of molecular pentafoil knots (5_1_) via circular double helicates. a) Fe_5_ **44** is obtained by formation of an imine bond between ligand **42** and diamine **43** in the presence of Fe^II^. The size (pentamer) of the circular helicate is determined by a chloride anion template.^105^ b) The yield of the pentafoil knot is increased by using ligand **45**, which allows for covalent capture of the closed‐loop knot by RCM. In contrast to Fe_5_ **44**, Fe_5_ **46** does not decompose upon demetalation.^107^ c) Solid‐state structure of Fe_5_ **46** (the structure of Fe_5_ **44** was also determined by X‐ray crystallography).

Circular helicate systems of the form Fe^II^_*x*_L_*x*_ with *x*=4, 5, and 6 were serendipitously discovered by Lehn and co‐workers in the 1990s.^102^, ^103^, ^104^ The value of *x* is affected by the ligand structure and an anion template effect. The ligands form a double helix woven around the metal centers, which means that, in principle, suitably modified ligand strands could be used to form torus knots and links. The first knot to be synthesized from a circular helicate was pentafoil knot (5_1_) **44**, which was assembled by formation of imine bonds^105^ (Scheme 17 a). The diamine building block **43** contains two oxygen atoms that allow the required folding of the glycol chain because of the *gauche* effect.^106^ Pentafoil knot **44** could not be demetalated due to the lability of the imine groups when not coordinated to a metal center. The related pentafoil knot **46** (formed from alkene‐terminated ligand **45**) was covalently captured by RCM in 98 % yield (Scheme 17 b), and could be readily demetalated under basic conditions.^107^

The X‐ray crystal structures of both **44** and **46** (the latter is shown in Scheme 17 c) feature a chloride ion originating from the assembly process present in the central cavity. A Solomon link^108^ (a doubly interlocked [2]catenane) and a Star of David catenane^109^ (a triply interlocked [2]catenane) have also been synthesized by using this approach through the use of tetrameric (*x*=4) and hexameric (*x*=6) circular helicates, respectively.

### 3.4 Higher Order Knots

**Figure 20:**
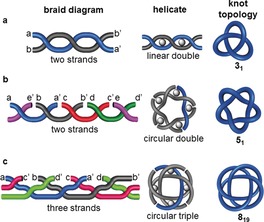
The complexity of knots accessible from helicates increases from a) a linear double helicate to b) a circular double helicate to c) a circular triple helicate.^110^

Recently the circular helicate strategy was successfully extended from double to triple helicates. This was possible because the Fe^II^ ions used to assemble the circular helicates are octahedral, and so can organize three strands containing bidentate groups, rather than only two. In braid representations, this means changing the braid from the one depicted in Figure 10 a to the one in Figure 12 a, as shown in Figure 20.

**Scheme 18:**
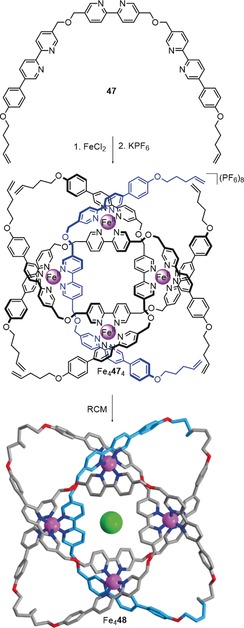
Synthesis of an 8_19_ knot based on the circular triple helicate approach. Tetramer Fe_4_ **47**_4_ is formed by the reaction of ligand **47** with FeCl_2_. Covalent capture by RCM yields knot Fe_4_ **48**.^110^.

A molecular 8_19_ knot was prepared by using this approach (Scheme 18); the resulting structure is the tightest knot reported to date, with 24 atoms per crossing.^110^ The reaction of ligand **47** with FeCl_2_ generated a circular triple helicate, which was closed to the 8_19_ knot through RCM. Steric restraints made sure that the closures could only take place between strands coordinated to neighboring iron centers, thereby affording the non‐alternating molecular 8_19_ knot **48**. This method for connecting strands that are not bound to the same metal center should be applicable to a range of higher order knots and links.

### 3.5 Composite Knots

**Figure 21:**
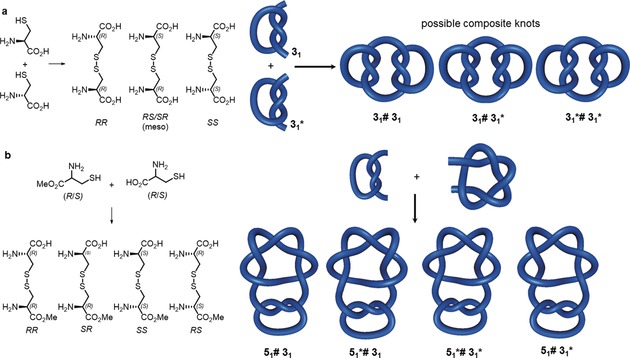
Synthesizing composite knots by forming the knot sum. a) Forming the homodimer of a chiral knot can result in two enantiomeric knots of opposing handedness (if two knots of identical chirality are joined) and one achiral knot (by joining knots of opposing handedness). This is analogous to forming dimers of a racemic compound: a *meso* diastereomer (combining *R* and *S*) is obtained as well as chiral diastereomers (combining *R* and *R* or *S* and *S*). b) Forming the knot sum of two different chiral knots gives four distinguishable knots, analogous to joining two different chiral centers in a molecule.

Small‐molecule composite knots (see Section 2.3) have yet to be synthesized as discrete entities. Their synthesis is rendered difficult by the fact that the combination of two chiral knots can give multiple products (Figure 21). Just as the dimerization of a racemic chiral molecule can give two enantiomeric chiral dimers (*RR* and *SS*) and an achiral *meso* compound (*RS*), the same is true for knots (Figure 21 a): The connected sum of a chiral knot with itself can form two enantiomeric knots (such as when two left‐handed or two right‐handed trefoil knots are connected to form granny knots) and one achiral knot (such as when two trefoil knots of opposite handedness are connected to form a square knot). If two different chiral knots are connected, four different combinations are possible (Figure 21 b). The knot sum of two achiral knots always yields an achiral composite knot.^111^, ^112^

**Scheme 19:**
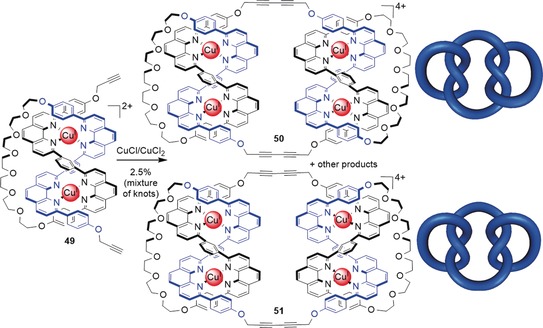
Synthesis of a mixture of molecular composite knots. The dimerization of two open trefoil knots **49** leads to the formation of molecular granny knot **50** and molecular square knot **51** among other products, as deduced by MS and NMR data. Which knot is formed is determined by the handedness of the two open trefoil precursors. If two complexes of the same handedness are combined, a granny knot is obtained, while the combination of two complexes of opposing handedness yields a square knot.^113^

To date, progress on the synthesis of small‐molecule composite knots is limited to the report by Sauvage and co‐workers of the low‐yielding synthesis of a mixture of composite knots. The dimerization of racemic open trefoil knot precursor **49** by Glaser coupling gave trace amounts of a product that was assigned to be a mixture of granny and square knots **50** and **51** (Scheme 19).^113^

## 4 Properties and Applications of Small‐Molecule Knots

Knotting a molecular backbone significantly restricts the conformations a molecule can adopt, effectively preorganizing the structure through mechanical constraints. It can induce chirality, irrespective of the presence of classical Euclidean stereochemical elements. Although the number of synthesized knots is still small, small‐molecule knots have already been shown to exhibit strong and selective anion binding, chirality, and catalytic activity, including asymmetric catalysis and allosteric catalysis.

### 4.1 Knot Dynamics

The dynamic properties of catenanes and rotaxanes have been explored as part of the development of artificial molecular machines.^114^, ^115^ The dynamic behavior of the mechanically constrained backbones of molecular knots is, as yet, relatively unexplored. In their seminal paper on molecular trefoil knots,^27^ Dietrich‐Buchecker and Sauvage reported that demetalation of the knot leads to broadening of the aromatic region of the ^1^H spectrum, thus suggesting slow reptation (snake‐like movement) of the knot chain. This effect was not observed for non‐interlocked side products. Similar broadening was observed in the recently synthesized 8_19_ molecular knot, which is particularly tightly knotted.^110^ It was subsequently shown that removing just a single metal ion from Sauvage's trefoil knots results in a conformational change which, depending on the spacer used in the helicate, rendered removal of the remaining metal ion either faster (with an alkyl linker) or slower (with a *m*‐phenylene linker).^116^ Lukin and Vögtle reported that the dynamic behavior of his hydrogen‐bonded trefoil knots is solvent‐dependent; in solvents other than DMSO, the knots were found to undergo slow dynamic motion (indicated by broad signals in the ^1^H NMR spectra).^117^

### 4.2 Chirality

**Figure 22:**
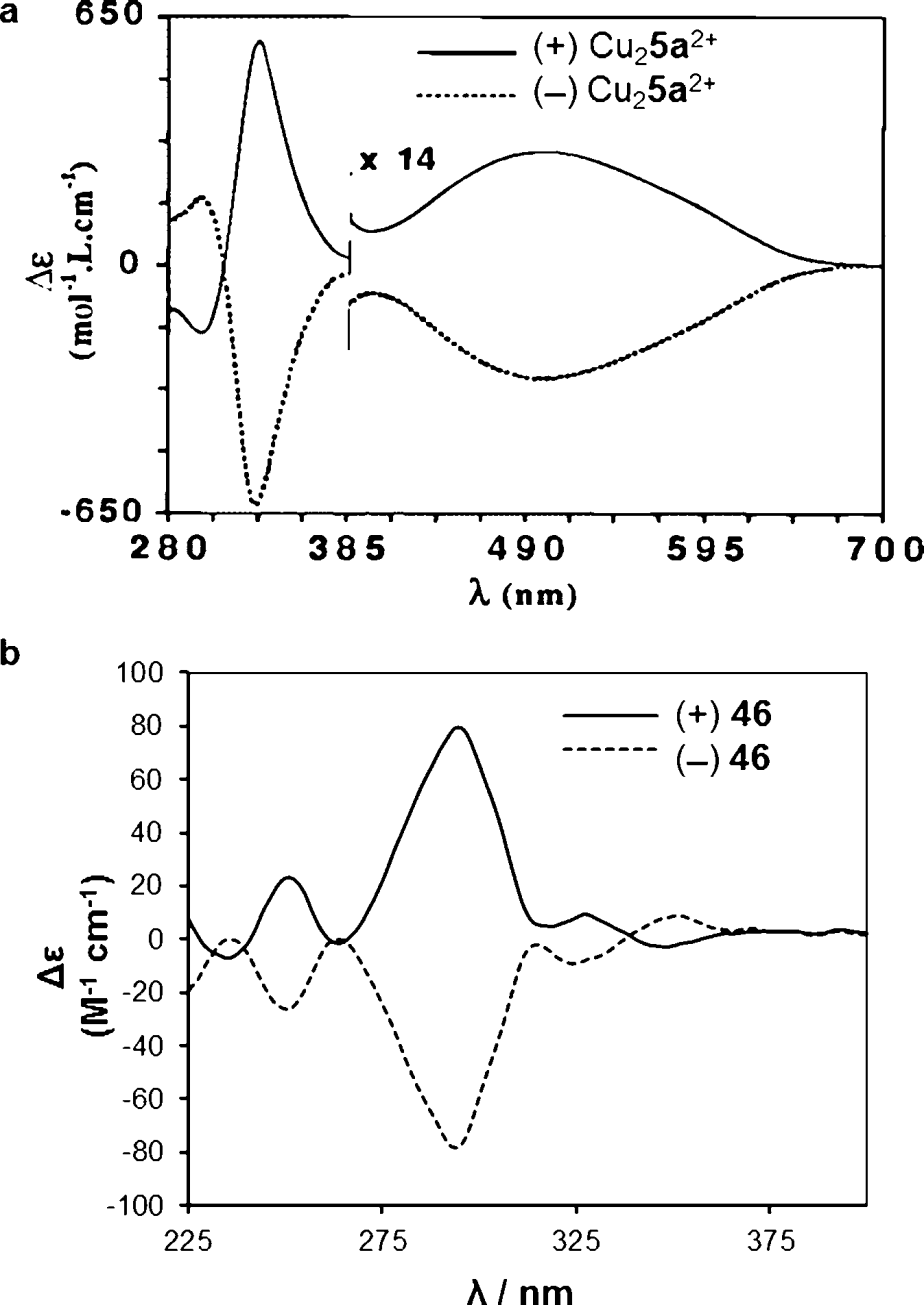
Circular dichroism (CD) spectra of the two enantiomers of a) Sauvage's trefoil knot **5 a** 67 and b) pentafoil knot **46**.^107^ Neither knot has elements of Euclidean chirality. Reproduced from Refs. 67 and 107 with permission from Wiley‐VCH and the American Association for the Advancement of Science, respectively.

Some examples of topological chirality in small‐molecule knots have been studied, as it is possible to either carry out the synthesis of chiral knots asymmetrically^68^, ^73^, ^74^, ^84^, ^87^, ^99^ or separate the enantiomers of knots produced through a racemic synthesis.^64^, ^67^, ^83^, ^107^, ^110^ The enantiomers of chiral knots have been studied by circular dichroism (Figure 22), and the spectrum of enantiopure trefoil knot **15 b** shows a greater ellipticity than the corresponding topologically isomeric macrocycle. This finding suggests that the topological chirality of the knot has a significant effect on the asymmetry of the chromophore environment.^73^

**Figure 23:**
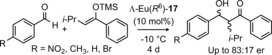
Asymmetric catalysis of the Mukaiyama aldol reaction by a chiral trefoil knot.^74^

Enantiopure trefoil knot **17**, whilst encapsulating europium, was found to catalyze the asymmetric Mukaiyama aldol reaction with up to 66 % *ee* (Figure 23).^74^ On the basis of luminescence decay lifetime measurements, it was postulated that the mechanism of the catalyzed reaction involved coordination of the aldehyde to the knot‐bound lanthanide ion whilst the knot maintained a chiral environment in the vicinity of the aldehyde.

### 4.3 Host–Guest Chemistry and Catalysis

Various small‐molecule knots have been found to act as host molecules that strongly bind to guest metal ions (which often facilitate their synthesis), organic molecules, or anions. Lukin and Vögtle found that a thin layer of an organic trefoil knot could adsorb octane.^117^ The trefoil knots of Trabolsi and co‐workers (Scheme 10) can be transmetalated and the anion within the central cavity changed (binding of I^−^, N_3_^−^, SCN^−^, and NO_3_^−^ reported).^90^, ^91^ Chloride anions are used to template the assembly of the pentameric circular helicates used to assemble 5_1_ knots **44** and **46** in Section 3.3.2. The resulting pentafoil knots bind chloride anions in the central cavity with *K*≈10^10^ m ^−1^ in MeCN, thus making them amongst the strongest chloride‐binding synthetic molecules known and with an affinity to chloride comparable to that of silver salts.^118^

**Figure 24:**
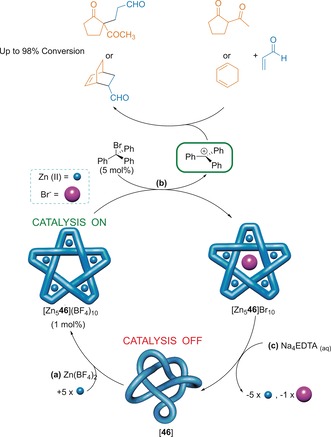
Allosteric regulation of Lewis acid carbocation catalysis by a molecular pentafoil knot. a) Remetalation of the knot **46** with Zn^2+^ gives the metalated knot with an empty central cavity. b) The metalated knot can remove bromide from trityl bromide to give the catalytically active trityl cation. c) Subsequent demetalation regenerates the organic knot ligand **46**, thereby shutting down the catalytic activity.^107^

Transmetalation of pentafoil knot **46** with Zn^2+^ allowed a derivative of the knot to be used for allosteric regulation of Lewis acid carbocation catalysis of Diels–Alder and Michael reactions (Figure 24).^107^ With the Zn^2+^ coordinated to the knot, a bromide ion could be abstracted from trityl bromide to yield a catalytically active trityl cation. No catalytic activity was observed when the knot was not present or was demetalated. In addition, the metalated knot could catalyze the Ritter reaction, whereby bromide was abstracted from bromodiphenylmethane to give the benzhydryl cation, which subsequently reacted with acetonitrile. The bromide ion was removed from the knot cavity by reaction with methyl triflate, thus regenerating the empty central cavity and allowing the catalyst to turn over.

In many of these early examples of properties and putative applications, the knotted architecture of the molecules plays a crucial role. With no elements of Euclidean chirality, it is the topology of the knot that leads to the CD response of **5 a** and **46** in Figure 22. Similarly, the knotted topology of ligand **46** is crucial for the allosteric catalysis shown in Figure 24; unknotted ligand strands coordinate with Zn^2+^ ions to form triple helicates and linear oligomers which do not bind anions nor have catalytic activity.

## 5 Synthetic and Biological Macromolecular Knots

### 5.1 Knots in Synthetic Polymers

**Figure 25:**
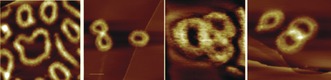
Knotted and interlocked cyclic polymers imaged by AFM.^125^ Adapted from Ref. 125 with permission from Wiley‐VCH.

Just as earphone cables and spaghetti have a tendency to become entangled, polymer chains of sufficient length and flexibility also undergo spontaneous knotting at the molecular level.^119^ The occurrence of knots in polymers was statistically modeled by Vologodskii and co‐workers, who predicted that knot formation was likely to occur in polymers where the length of the monomer unit was significantly longer than the thickness of the chain, such as DNA.^16^ Further studies determined that ever more complicated knots are likely to be formed as the length of a polymer increases, and that these knots are increasingly likely to be composite (see Section 2.3) rather than prime.^120^ Simulations have also shown that some knots are favored structures that could self‐assemble from solutions of helical building blocks with sticky ends.^121^ The location of knots in non‐uniform polymers has also been investigated.^122^, ^123^, ^124^ Recent investigations have allowed polymeric knots to be visualized directly by atomic force microscopy (AFM; Figure 25)^125^ and for their controlled assembly by metal‐template synthesis and high dilution cyclization.^126^, ^127^

**Figure 26:**
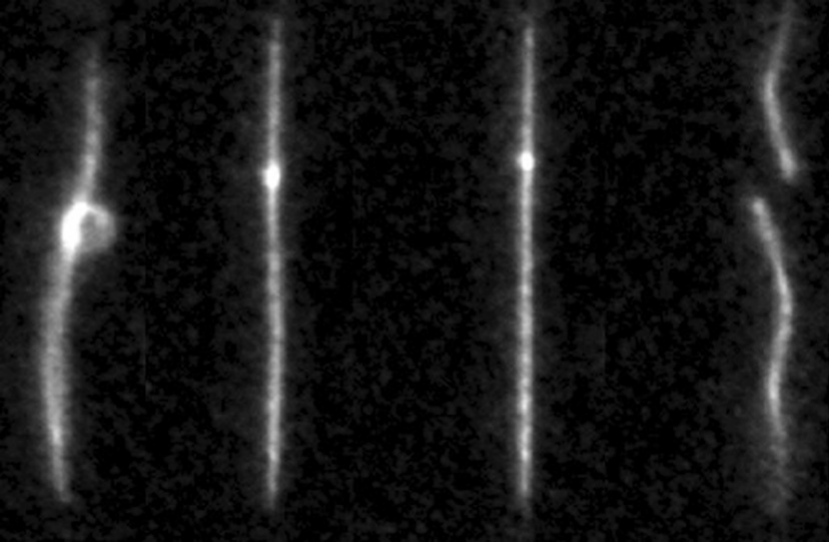
An actin filament tied in a knot by molecular tweezers.^129^ Tightening of the knot eventually causes the filament to break at the location of the knot. Adapted from Ref. 129 with permission from Springer Nature.

It is well known that knotting a string (or rope) decreases its strength, and when pulled at either end such a string will break at the entrance to the knot. Mountaineers and fishermen know to use knots with a high efficiency (i.e. a knot that decreases the strength of a strand the least) to maintain the strength of a rope or line.^17^ The weakening effect of tying a knot in a molecular strand has also been considered; it was shown theoretically that tightening a knotted polyethylene chain should cause strain energy to be located at the C−C bonds at the entrance to the knot.^128^ Further tightening of the knot might, therefore, result in the breaking of one of these C−C bonds at a lower dissociation energy than an unknotted chain. An experimental demonstration of such a phenomenon has been reported: tying a knot in an actin filament by using molecular tweezers reduced its tensile strength by approximately two orders of magnitude, and pulling of the polymer chain caused breakage where the knot was located (Figure 26).^129^

Additionally, intermolecular entanglement of multiple polymer chains can also result in knotting, thereby affecting the morphology and mechanical properties of polymeric materials.^130^ A related 3D interwoven material has recently been reported, which also displays high elasticity when demetalated.^131^

### 5.2 Knots in DNA

**Figure 27:**
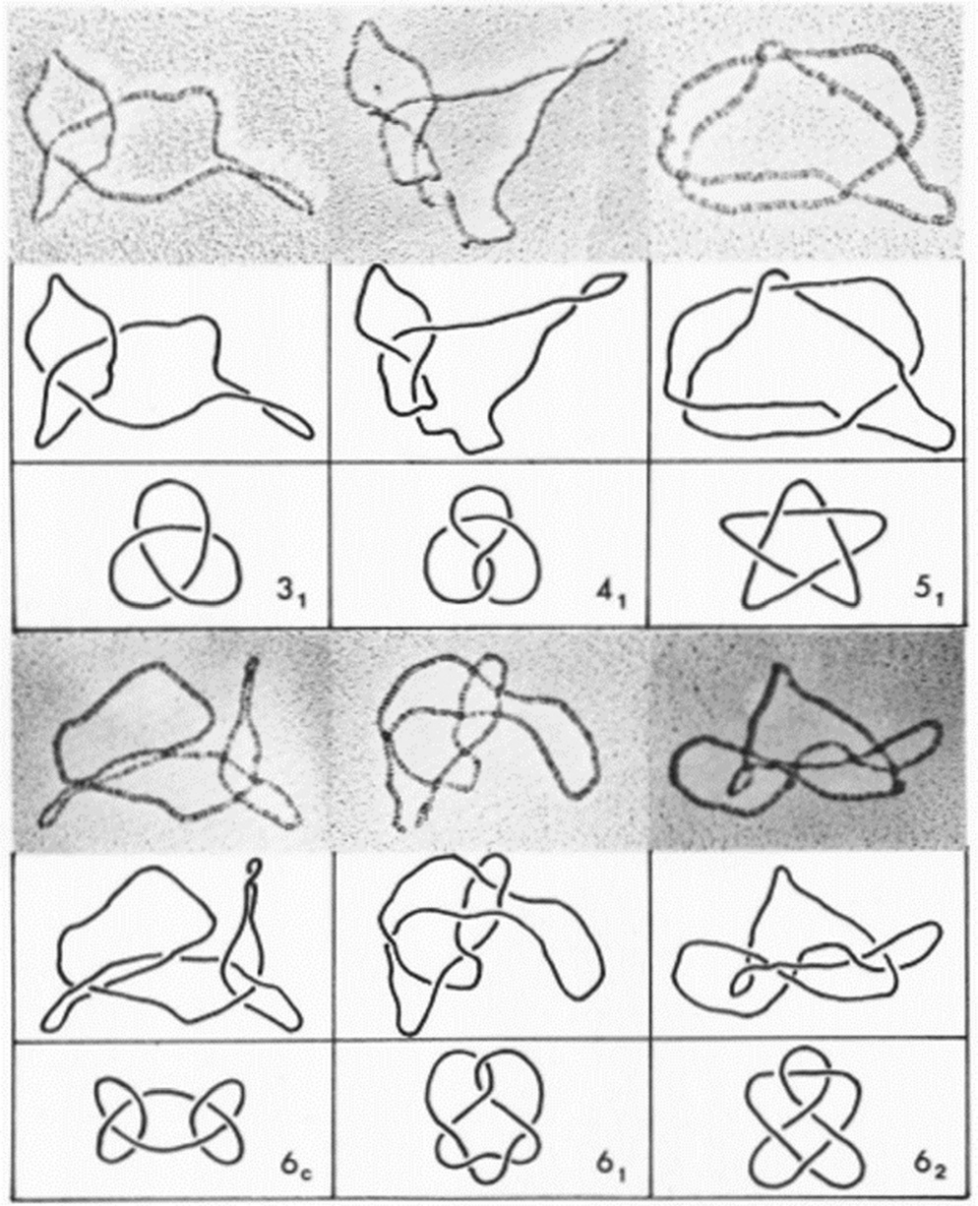
Knotted DNA (with conventional representations of knots) produced on incubation of circular DNA with a topoisomerase I enzyme and imaged by electron microscopy.^136^ Adapted from Ref. 136 with permission from the American Society for Biochemistry and Molecular Biology.

Examples of circular DNA containing knots were first found in 1976,^132^ nearly a decade after the discovery of naturally occurring DNA links.^133^, ^134^ The topology of the DNA was imaged by electron microscopy. DNA knotting is mediated by topoisomerase enzymes, which either allow the passage of a single strand through the nick in the complementary strand (Type I) or the passage of a segment of duplex DNA through a double‐stranded break (Type II).^135^ Incubation of DNA with topoisomerase I from *E. coli*. gave a mixture of all possible knots up to at least seven crossing points (with no stereocontrol, Figure 27).^136^

Topoisomerases were originally thought to act under thermodynamic control; however, topoisomerase II was shown to use ATP hydrolysis to reduce DNA knotting below the equilibrium value.^137^ The presence of knots in DNA hinders transcription and replication and can lead to mutations.^138^, ^139^, ^140^ Therefore, topoisomerases are required to maintain cell function by unknotting DNA and reducing supercoiling.^141^, ^142^ A molecule of circular DNA cannot be unknotted if topoisomerase enzymes are not present, unless its backbone is broken. Therefore, the knot invariant (see Section 2.1) of DNA does not change in routine manipulation (e.g. in isolation and analysis) and provides a useful handle for studying DNA in vitro.^14^

Supercoiled DNA can become knotted and form links during site‐specific recombination when genome rearrangement is performed by the recombinase enzymes.^143^ Tangle theory (Section 2.11) has proved useful in understanding the topological implications of the actions of such recombinases.^144^

Linear DNA is densely packed and highly confined in phage capsids, which results in a high writhe value (see Section 2.1).^145^ This manifests itself in a very high level of DNA knotting (ca. 95 % of the molecules), with a preference for torus knots (with a high writhe value) and only trace amounts of the achiral figure‐eight knot (with a very low writhe value) being observed.^146^

Perhaps surprisingly, naturally knotted RNA has not yet been reported, with the underlying reasons for its absence unclear.^147^ Artificial DNA and RNA knots have also been reported, through pioneering work by the Seeman group.^148^

### 5.3 Knots in Proteins

As most proteins consist of a backbone with two termini, they do not form the closed loops required for mathematically defined knots. However, imaginary connecting of the termini without generating additional crossing points provides a framework that allows entanglements (“knotting”) within proteins to be analyzed. In addition, cross‐linking of a protein backbone by disulfide bonds or prosthetic groups can lead to interlocked and knotted structures within proteins (e.g. “cysteine knots”).^149^, ^150^, ^151^

**Figure 28:**
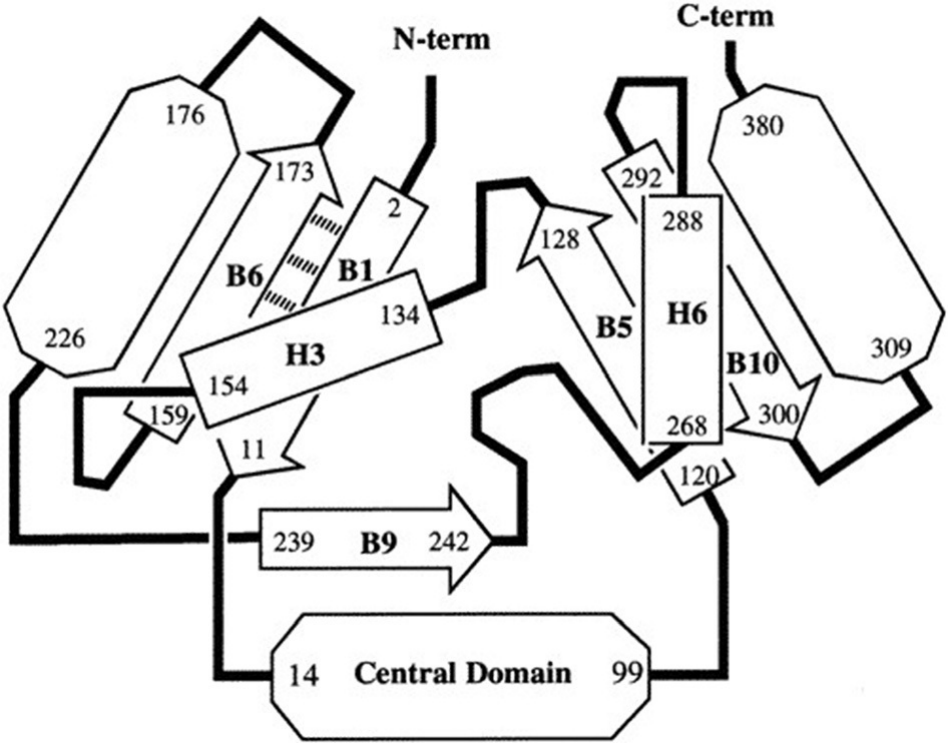
Schematic representation of a loosely knotted protein.^154^ A trefoil knot is formed by passage of the B9 β‐strand leading to the C‐terminus through a loop formed by the sequence B1→[central domain]→B5→H3→B6. Adapted from Ref. 154 with permission from the American Chemical Society.

The first protein to be identified with a knotted backbone was carbonic anhydrase.^152^ This protein is tied in a loose trefoil knot, but only a few residues need to be removed from one terminus to unknot the protein, so it was suggested that this protein only forms an “incipient” knot.^153^ It was suggested that the mechanisms of folding prevented reptation of the protein chain required for knot formation within the protein core. Another loosely knotted protein was reported shortly afterwards, also containing a trefoil knot, where up to 10 residues could be removed before unknotting the protein chain (Figure 28).^154^

**Figure 29:**
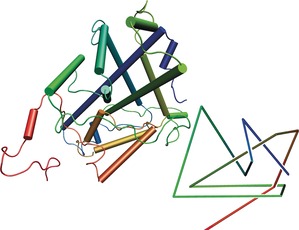
X‐ray crystal structure and reduced schematic diagram of DehI, the most complicated knotted protein known to date, which contains a Stevedore knot (6_1_).^156^ Adapted from Ref. 156 with permission from PLOS.

In 2000 Taylor introduced a new computational method for probing knots in proteins.^155^ This allowed entanglements buried in the core of proteins (known as deeply knotted proteins) to be discovered for the first time. Analysis of the Protein Data Bank (PDB) revealed a series of proteins that formed deeply knotted left‐ and right‐handed trefoil knots (3_1_) and two proteins that formed a more complicated figure‐eight knot (4_1_). One of the latter proteins, acetohydroxy acid isomeroreductase, required the removal of over 300 residues to unknot the protein. Many other knots in proteins have been identified, with the most complicated to date being a Stevedore knot (6_1_) in DehI, an α‐haloacid dehalogenase (Figure 29).^156^

A recent analysis of the PDB, however, suggests that the proportion of proteins that are knotted is small (ca. 1 %).^157^ This proportion is lower than what would be expected for similar heteropolymers, which implies that nature has, for the most part, specifically avoided protein knotting.^158^ Simulations have suggested that local ordering within the hydrophobic core of proteins disfavors entanglement and knot formation, which itself is determined by the protein sequence.^159^ Interestingly, it has also been found that many knotted proteins have loop segments, which unknotted proteins with similar structures or sequences lack, and so may be required to promote knotting.^160^ This suggests that the knotting of proteins may be at least partially encoded in protein primary structure. However, a study of the first synthetic knotted protein, whose sequence was derived by modifying an unknotted dimeric protein, showed it could fold in seconds without the need to incorporate knot‐promoting segments.^161^

Although proteins may not necessarily contain a knot for a specific purpose, it is worth noting that ubiquitin hydrolase needs to be particularly resistant to unfolding. It folds to give a 5_2_ knot, and it has been shown that complex knotting grants kinetic stability to proteins.^162^, ^163^ As most known knotted proteins are enzymes, and the knot is usually located in the catalytic domain, knots may have an important effect on enzymatic activity.^164^, ^165^ The presence of a cysteine knot within the core of a protein has been shown to confer exceptional stability.^166^

## 6 Conclusions and Outlook

The past 30 years have seen the invention and serendipitous discovery of a range of synthetic strategies for the construction of molecular trefoil knots, enabled by progress in the control of reactivity, conformation, and supramolecular structure. However, the synthesis of other molecular knots remains an almost entirely unconquered challenge for synthetic chemistry. Of the six billion prime knots tabulated to date,^29^ only four—the trefoil, figure‐eight, pentafoil, and 8_19_ knot—have been synthesized thus far using small‐molecule building blocks. That is a vast volume of completely unexplored molecular space. As the number of molecular knot topologies that become accessible increases, chemists will start to develop a fuller picture of their properties (at which point are entangled molecular strands prone to breaking? and if knotting weakens a molecular strand, can it be used to promote bond breaking?) and will discover which knots have properties best suited for a particular purpose. Once we can make molecular knots and understand their properties, knotting may start to have an impact on functional molecule and material design in the same way that tying knots proved so important for advancing the technology of our earliest ancestors.

## 7 Glossary

An explanation of knot, braid, and tangle terminology used in this Review that may be unfamiliar to chemists:

Achiral/amphichiral/amphicheiral knot: A knot that can be deformed continuously into its mirror image.
Alexander–Briggs notation: Notation of the form *X_Y_* used to distinguish a knot from others. *X* refers to the number of crossings, *Y* is a variable used to differentiate knots with the same number of crossings.
Alternating knot: A knot that can be represented in a way that over‐ and underpasses of the strand alternate.
Braid representations: Every knot can be represented as a braid of *n* strands. The closed loop knot is obtained by connecting the strands at the braid ends (see Section 2.10).
Chiral knot: A knot that cannot be continuously deformed into its mirror image.
Clasp knot: A generalization of a twist knot (see Section 2.8).
Composite knot: A knot that can be described as the combination of two or more prime knots.
Crossing: A point in which the projection of a knot crosses itself; crossings can be positive or negative (see Section 2.1).
Gordian distance: The number of crossing changes needed to interconvert two knots (see Section 2.12).
Invariant: An intrinsic property of a particular knot, for example its minimum number of crossings.
Invertible knot: A knot that can be continuously deformed into a reversed orientation of itself (see Section 2.5).
*k*‐moves: The introduction of *k* positive crossings into a set of two strings.
Non‐alternating knot: A knot that cannot be represented in a way where over‐ and underpasses alternate.
Nugatory crossing: A crossing that can be removed by twisting.
Pretzel knots: Knots obtained by connecting left‐ and right‐handed helices to form closed loops (see Section 2.11).
Prime knot: A knot that cannot be described as the combination of simpler knots.
Reduced representation: Depiction of a knot with its minimum number of crossings.
Reidemeister moves: A set of string manipulations that transform different representations of the same knot into each other (see Section 2.1).
Tangles: Building blocks of entanglements from which knots can be created.
Torus knot: A knot that can be drawn on the surface of a torus without intersecting (see Section 2.7).
Twist knot: A knot created by twisting two strands *n* times and interlocking the open ends before closure (see Section 2.8).
Unknot (trivial knot): A knot that can be deformed into a representation without any crossings.
Unknotting number: The minimum number of crossings of a knot that have to be flipped to yield the unknot.
Writhe: The sum of positive and negative crossings in the representation of a knot (see Section 2.1).

## Conflict of interest

The authors declare no conflict of interest.

## Biographical Information

*Stephen Fielden was born in Bury, Greater Manchester (UK). He obtained his MChem from the University of Oxford in 2015 and then joined Prof. David Leigh's group for a PhD funded by a Dean's Award from the University of Manchester. His research interests include the development of complex molecular topologies and novel methods of controlling molecular motion*.

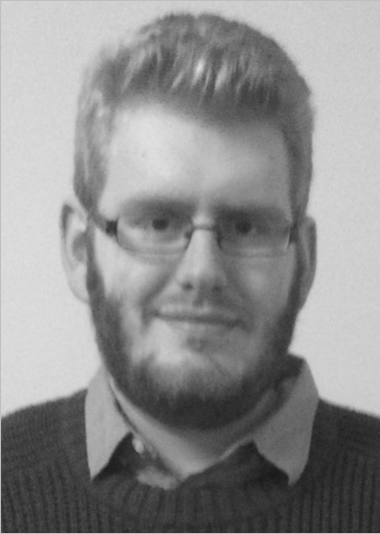

## Biographical Information

*David Leigh obtained his BSc and PhD from the University of Sheffield. After postdoctoral research in Ottawa (1987–1989) he was appointed to a Lectureship at the University of Manchester Institute of Science and Technology (UK). After holding Chairs at the Universities of Warwick and Edinburgh, he returned to Manchester in 2012, where he currently holds the Sir Samuel Hall Chair of Chemistry and is a Royal Society Research Professor. His research interests include chemical topology and synthetic molecular motors and machines*.

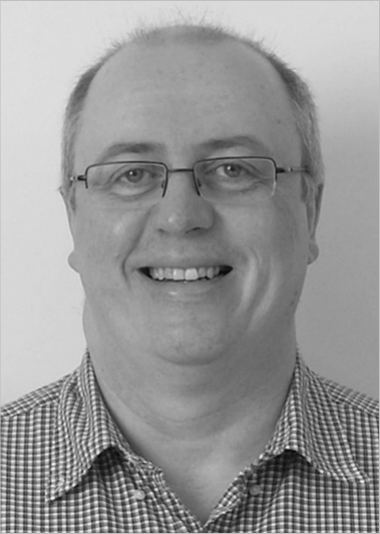

## Biographical Information

*Steffen Woltering was born in Münster (Germany). He studied chemistry at the Georg‐August‐Universität Göttingen (Germany) and the University of Edinburgh (UK), and obtained his MSc from the former in 2012. He recently completed his PhD in Prof. David Leigh's group in Manchester on the template synthesis of interlocked molecules*.